# Inhibition of Neuroblastoma Tumor Growth by Ketogenic Diet and/or Calorie Restriction in a CD1-Nu Mouse Model

**DOI:** 10.1371/journal.pone.0129802

**Published:** 2015-06-08

**Authors:** Raphael Johannes Morscher, Sepideh Aminzadeh-Gohari, René Gunther Feichtinger, Johannes Adalbert Mayr, Roland Lang, Daniel Neureiter, Wolfgang Sperl, Barbara Kofler

**Affiliations:** 1 Research Program for Receptor Biochemistry and Tumor Metabolism, Paracelsus Medical University, Salzburg, Austria; 2 Department of Pediatrics, Paracelsus Medical University, Salzburg, Austria; 3 Department of Dermatology, Paracelsus Medical University, Salzburg, Austria; 4 Department of Pathology, Paracelsus Medical University, Salzburg, Austria; 5 Division of Medical Genetics, Medical University Innsbruck, Innsbruck, Tirol, Austria; Duke University Medical Center, UNITED STATES

## Abstract

**Introduction:**

Neuroblastoma is a malignant pediatric cancer derived from neural crest cells. It is characterized by a generalized reduction of mitochondrial oxidative phosphorylation. The goal of the present study was to investigate the effects of calorie restriction and ketogenic diet on neuroblastoma tumor growth and monitor potential adaptive mechanisms of the cancer’s oxidative phosphorylation system.

**Methods:**

Xenografts were established in CD-1 nude mice by subcutaneous injection of two neuroblastoma cell lines having distinct genetic characteristics and therapeutic sensitivity [SH-SY5Y and SK-N-BE(2)]. Mice were randomized to four treatment groups receiving standard diet, calorie-restricted standard diet, long chain fatty acid based ketogenic diet or calorie-restricted ketogenic diet. Tumor growth, survival, metabolic parameters and weight of the mice were monitored. Cancer tissue was evaluated for diet-induced changes of proliferation indices and multiple oxidative phosphorylation system parameters (respiratory chain enzyme activities, western blot analysis, immunohistochemistry and mitochondrial DNA content).

**Results:**

Ketogenic diet and/or calorie restriction significantly reduced tumor growth and prolonged survival in the xenograft model. Neuroblastoma growth reduction correlated with decreased blood glucose concentrations and was characterized by a significant decrease in Ki-67 and phospho-histone H3 levels in the diet groups with low tumor growth. As in human tumor tissue, neuroblastoma xenografts showed distinctly low mitochondrial complex II activity in combination with a generalized low level of mitochondrial oxidative phosphorylation, validating the tumor model. Neuroblastoma showed no ability to adapt its mitochondrial oxidative phosphorylation activity to the change in nutrient supply induced by dietary intervention.

**Conclusions:**

Our data suggest that targeting the metabolic characteristics of neuroblastoma could open a new front in supporting standard therapy regimens. Therefore, we propose that a ketogenic diet and/or calorie restriction should be further evaluated as a possible adjuvant therapy for patients undergoing treatment for neuroblastoma.

## Introduction

Neuroblastoma (NB) is the most common extra-cranial solid malignancy of childhood. This tumor of the peripheral nervous system originates from primitive sympathogonia that normally give rise to the postganglionic neurons of the sympathetic chain and the adrenal medulla [1]. Based on biological and clinical features, the disease can be classified into three risk categories. In the high-risk patient population, despite extensive efforts to improve treatment strategies, the prognosis is poor, with an estimated 5-year survival of 50%. This contrasts to an overall survival rate exceeding 90% in the intermediate- and low-risk groups, where treatment optimization has shifted toward reducing the toxicities of the multi-modal therapy approach [2].

The mitochondrial energy metabolism of NB is characterized by generalized low protein and activity levels of the oxidative phosphorylation (OXPHOS) complexes, along with a low copy number of the mitochondrial genome (mtDNA). Mitochondrial mass, however, as quantified by markers such as citrate synthase activity and voltage-dependent ion channel (VDAC) protein content, is similar to that of normal adrenal gland and kidney tissues [3]. Although the mechanisms inducing this phenotype are still under investigation, data indicate that it is part of the metabolic reprogramming of NB cells that permits the state of low differentiation and high proliferative capacity. Changes in mitochondrial function have been shown to be a central component of the induction of differentiation in NB cell lines. Retinoic acid treatment, for instance, significantly increases basal oxygen consumption as well as respiratory capacity [4–6].

These observations are in line with the reprogramming of cancer cell metabolism to increased glucose utilization, one of the hallmarks in cancer development [7–9]. The dependency of cancer cells on anaerobic glycolysis, even when there is sufficient oxygen available to shunt pyruvate into the OXPHOS pathway, is widely known as the Warburg effect [10]. Isolated defects in enzymes of the OXPHOS system can be a direct cause of cancer formation and the Warburg effect, as exemplified in pheochromocytomas and paragangliomas (both with defects in complex II) or oncocytomas (defects in complex I). Other solid tumors such as renal cell carcinomas or NBs are characterized by a more general reduction of all OXPHOS complexes [3, 11–18].

Cancer therapies have always employed approaches targeting phenotypic characteristics of cancer cells that render them more sensitive to a specific treatment than the rest of the body’s cells. Similarly, in radiology, the increased uptake of glucose by cancer cells is widely employed in ^18^F-fluorodeoxyglucose positron emission tomography [19]. In contrast, metabolism targeted chemotherapeutics available for clinical application are mainly limited to nucleic acid metabolism (i.e. 5-fluorouracil and methotrexate) and asparaginase treatment [20]. Therapeutic targeting of the glucose-dependency of cancer cells has long focused on the (preclinical) use of 2-deoxyglucose, a compound that is thought to block glycolytic flux. However, this once hopeful approach has failed to translate to the clinic due to its toxicity at high doses, although it is still being evaluated at lower doses [21, 22]. Alternative substances that broaden the metabolic targets from glycolysis to lipid- and amino acid metabolism or the tricarboxylic acid cycle are under evaluation [20, 22–25]. Additionally, the adaptation of dietary intake to reduce side effects and/or support cancer therapy is being revisited in pre-clinical and clinical studies. Changing the patient’s nutrition to a ketogenic diet (KD), for instance, is thought to reduce the growth of tumors of the central nervous system (CNS) by shifting abundant blood substrate levels from glucose to ketone bodies and fatty acids [26–33].

KD with or without calorie restriction (CR) has for decades been part of the management of children with intractable seizures [34]. In the pediatric population, KD is widely tolerated without major side effects and has gained attention in treating adolescent and adult therapy-resistant epilepsy [35]. Classic regimens (such as the Johns Hopkins protocol) offer a food-content ratio of fats to carbohydrates and proteins of 3:1 or 4:1 (in grams). Adaptations to this basic regimen such as combining it with variable CR or more liberal approaches such as the modified Atkins diet are commonly applied [36, 37]. More recently, KD has been under evaluation for alternative indications such as obesity, polycystic ovarian syndrome and degenerative diseases of the nervous system [38]. The molecular mechanism of KD is poorly understood but data point toward multifactorial direct and indirect metabolic adaptations, including increased mitochondrial biogenesis and an altered cellular energy state [36, 39].

Preclinical data on cancers of the CNS suggest that CR and/or KD can be a potential adjuvant approach in cancer therapy [31, 40–42]. This is supported by two case reports of patients treated with CR-KD [43, 44], but extensive clinical evaluation is lacking. Two pilot studies of KD in 36 adult patients showed that KD can be tolerated in patients with advanced cancers [28, 30]. We recently reported that astrocytomas show a grade-dependent loss of mitochondrial respiratory chain activity, further substantiating the glucose-dependency and therefore the likely susceptibility to KD of high-grade neuroepithelial tumors [45]. Our laboratory systematically characterized OXPHOS activities in a range of human cancers, since functional data on the OXPHOS system in different cancer types are sparse [3, 11–18]. In the present study we chose NB as a model tumor because NBs show a comparable reduction in OXPHOS as neuroepithelial tumors of the CNS [3, 45]. Moreover, in vitro data indicate that NB cells preferentially use glucose over ketone bodies as an energy source [46] and that ketone bodies potentially reduce the viability of NB cells [47]. In the context of this information, we designed a study to evaluate the effects of dietary intervention on the growth of NB xenografts and to monitor potential adaptations of the OXPHOS system.

## Methods

### Cell lines

The cell lines SH-SY5Y (ATCC CRL-2266) and SK-N-BE(2) (ATCC CRL-2271) were chosen for the xenograft studies. SH-SY5Y is a *TP53* wild-type, non-*NMYC*-amplified cell line that shows no chromosome 1p loss of heterozygosity and is sensitive to chemotherapy. The SK-N-BE(2) cell line shows high resistance to a wide range of chemotherapeutic agents and is characterized by *NMYC* amplification, *TP53* mutation (p.C135F) and chromosome 1p loss of heterozygosity [48]. Cells were grown in a 1:1 mixture of Eagle's Minimum Essential Medium and Ham’s F12 (Sigma), supplemented with 10% fetal bovine serum (PAA), Glutamax (Gibco), non-essential amino acids (Sigma) and penicillin/streptomycin/amphotericin (Sigma).

### Animal models and sample preparation

All *in vivo* experiments were performed in accordance with protocols approved for this study by the Salzburg Animal Care and Use Committee (Study approval No. 20901-TVG/44/7-2011). Animals were maintained under specific pathogen-free conditions and care conformed to the Austrian Act on Animal Experimentation. Xenografts were established by subcutaneous injection of a 200 μl matrigel (BD Bioscience) / serum-free medium suspension of NB cells (2.7×10^7^) on the right flank of 5- to 6-week-old female CD-1 nude mice (Charles River). After reaching a tumor size of 150 mm^3^, the mice were randomized to four dietary therapy groups (n = 8–11).

Tumor volumes were measured two times weekly using calipers and calculated with the formula width*height*length/2. Body weight, blood glucose and ketone body levels (beta-hydroxybutyrate) were monitored twice weekly after a two-hour fasting period in the ad libitum fed groups or before feeding in the calorie-restricted groups. The applied technique is widely used in clinical routine diagnostics and utilizes enzymatic based methods for quantification (Precision Xceed, Abbott) [49–51]. After inducing sevoflurane anesthesia mice were sacrificed by cervical dislocation at predetermined days (day 22 SH-SY5Y and day 33 SK-N-BE (2)) or when termination criteria were met (health status, tumor ulceration or volume of 3000 mm^3^). Cancer tissue was snap frozen in liquid nitrogen for OXPHOS measurements and determination of mtDNA copy number. One 0.5-cm tumor slice each was formalin-fixed and paraffin-embedded for histological analysis.

### Food composition and energy content

Mice were fed according to four different regimens: standard diet ad libitum (SD), calorie-restricted standard diet (CR-SD), long chain fatty acid based ketogenic diet ad libitum (KD) and calorie-restricted ketogenic diet (CR-KD). The metabolizable energy contents were as follows: SD (kcal: fat 9%, protein 33% and carbohydrates 58%) and long chain fatty acid based KD (kcal: fat 78%, protein 14% and carbohydrates 8%) (No. V1535-000 and No. S9139-E02D; Sniff Spezialdiäten GmbH). A detailed list of ingredients is given in Supporting Information (S1 Table). Diets were fortified with vitamins and mineral supplements and differed slightly between the two diets. To determine average calorie intake, 5 mice were monitored for ad libitum feeding for over a 5-week period (SD: mean 6.22 g ± 0.96 /d/mouse; KD: 2.7 ± 0.4 g /d/mouse). For the CR groups, food intake was restricted to 2/3 of the corresponding ad libitum intake. This degree of CR corresponds to those of clinical protocols for therapy-resistant epilepsy [35–37].

### Extraction of 600g homogenate

A mitochondria-enriched homogenate obtained by differential centrifugation was used for enzyme activity measurement and western blot analysis of OXPHOS complexes. This method increases specificity and is also used for the evaluation of inherited metabolic diseases [52, 53]. A tissue disintegrator (Ultraturrax, IKA) was used to homogenize NB xenograft tissue (50–100 mg) in SEKT-extraction buffer, pH 7.6 (250 mM sucrose, 2 mM EGTA, 40 mM KCl, 20 mM Tris-HCl). Cell membranes were then sheered with a motor-driven Teflon-glass homogenizer (Potter S, Braun). Centrifugation of the homogenate at 600g for 10 min at 4°C yields the post-nuclear supernatant (600g homogenate) containing the mitochondrial fraction.

### Enzyme measurements

Xenograft 600g homogenate of all cases in the SH-SY5Y groups (each n = 8–11) and of representative cases in the SK-N-BE(2) groups (each n = 5) were used for enzymatic measurements. Activities of all individual enzymes of the OXPHOS complexes I-V) and combinations (complexes I+III and complexes II+III) were measured as previously described [3, 11, 54]. In addition, CS activity was measured because it is a well-established marker of mitochondrial mass [55]. All quantifications were based on spectrophotometric measurements (Uvicon 922, Kontron) and performed at 37°C, with the exception of complex V, 30°C.

In brief: CS (EC 2.3.3.1) activity was recorded according to Srere [56] with modifications as noted in [3]. 10 μl of 600g homogenate was mixed with a Tris-HCl-buffered reaction mixture containing: 0.1% bovine serum albumin, 0.1% Triton X-100, 0.2 mM Elman’s reagent [5,5’-dithio-bis(2-nitrobenzoic acid)] and 0.15 mM acetyl-CoA. After recording thiolase activity for 2 min, the CS reaction was started by addition of 0.5 mM oxaloacetate and extinction dynamics were followed at 412 nm for 8 min. Rotenone-sensitive complex I activity was measured spectrophotometrically as NADH:decylubiquinone oxidoreductase (EC 1.6.5.3) at 340 nm. The enzyme activities of complex IV (ferrocytochrome c:oxygen oxidoreductase EC 1.9.3.1) and oligomycin-sensitive complex V ATPase (complex V, F_1_F_0_ ATP synthase, EC 3.6.3.14) were determined by using buffer conditions as previously described by Rustin et al. [57]. The final reaction mixture for the complex V activity measurement was treated for 10 bursts with an ultra-sonifier (Bio cell disruptor 250, Branson). The complex II (succinate dehydrogenase, succinate:ubiquinone-oxidoreductase EC 1.3.5.1) reaction mixture was pre-incubated for 10 min at 37°C. Quantification was performed for 6 min at 600 nm by monitoring the reduction of 2,6-dichlorophenol-indophenol after addition of 10 mM succinate. The reaction mixture for the measurement of the complex III activity (EC 1.10.2.2 coenzyme Q:cytochrome c—oxidoreductase) contained 100 μM cytochrome c and 200 μM reduced decyl-ubiquinol. The reaction was started by addition of the 600g homogenate. After 3–4 min the reaction was inhibited by addition of 1 μM antimycin A. Antimycin A-insensitive activity was subtracted from the total activity to calculate complex III activity. When not stated otherwise, reagents were obtained from SIGMA.

### Determination of the mtDNA copy number

For the determination of mtDNA copy number, proteinase K (Roche Diagnostics) treatment was performed before extraction of total DNA from tumor tissue [58]. Quantitative real-time PCR (iCycler, BioRad) was performed using two independent mitochondrial (m.6625_6754 and m.9910_10198) and nuclear DNA loci (*POLG* and *RRM2B*). Then, mtDNA content was determined by calculating the ratios of the Ct values. MtDNA copy number is given as n/diploid genome (the sequences of the primer-pairs 1–2 and 3–4 are given in S2 Table) [59].

### Western blot analysis

600g homogenates were used for western blot analysis as described before [3]. In brief, a total of 10 μg protein was separated on 10% acrylamide/bis-acrylamide gels and transferred to nitrocellulose membranes (Amersham Biosciences) using a CAPS buffer (10 mM 3-[cyclohexylamino]-1-propane sulfonic acid, pH 11; 10% methanol). The following primary antibodies were diluted in 1% western blocking reagent (Roche Diagnostics) dissolved in TBS-T: anti-GAPDH (Glyceraldehyde-3-phosphate dehydrogenase, 1:10000, Trevigen), anti-NDUFS4 (NADH dehydrogenase [ubiquinone] iron-sulfur protein 4; 1:2000, Abcam), anti-SDHA (Succinate dehydrogenase [ubiquinone] flavoprotein subunit; 1:5000, Abcam), anti-UQCRC2 (Cytochrome b-c1 complex subunit 2; 1:1000, Sigma), anti-MTCO2 (Cytochrome c oxidase subunit 2; 1:5000, Abcam), anti-ATP5A (ATP synthase subunit alpha; 1:1000, Protein Tech), anti-SCOT (Succinyl-CoA:3-ketoacid coenzyme A transferase 1; 1:1000, Abnova). Horseradish peroxidase-labeled secondary antibodies were used at a dilution of 1:1000 (Dako) and detection was carried out with Lumi-Light POD-substrate (Roche).

### Immunohistochemical staining and analysis

OXPHOS complexes (complex I-V) and proliferation indices (Ki-67 and PHH3) were evaluated in deparaffinized tumor sections of NB xenografts. For immunohistochemical staining the following antibodies were used: anti-NDUFS4 (1:1000, Abcam), anti-SDHA (1:2000, MitoSciences), anti-UQCRC2 (1:1500, MitoSciences), anti-MTCO2 (1:1000, MitoSciences), anti-ATP5A (1:2000, MitoSciences), and anti-VDAC1 (Voltage-dependent anion-selective channel protein 1; 1:3000, MitoSciences), anti-Ki-67 (1:200, Dako) and anti-PHH3 (1:500, Cellmarque). All antibodies were diluted in Dako antibody diluent with background-reducing components (Dako). The staining and scoring procedures for the OXPHOS complexes were performed as reported previously [17, 45]. Proliferation was scored by evaluating the proportion of positively stained nuclei, scoring at least 500 cells per slide (Ki-67) or counting the number of positively stained nuclei per high power field (PHH3). For all tumors at least five representative regions were scored, magnification was 400-fold.

### Statistics

Statistical analysis for continuous variables was performed by one-way ANOVA (p<0.05) and adapted for multiple comparisons by a two-tailed Dunnett’s post-test comparing each of the three therapy groups (CR-SD, KD and CR-KD) to the control (SD). Unless mentioned otherwise, the results are given as mean ± SEM. Survival is expressed by the Kaplan–Meier method and differences between groups were determined in a univariate analysis with the log-rank test. Western blot signal intensities were quantified using Image Lab 3.0.1. Statistical analysis was performed using Prism 5.03 (GraphPad Software) and SPSS 21 (IBM).

## Results

### Ketogenic diet and calorie restriction reduce tumor growth and prolong survival in mice with NB xenografts

**Fig 1:**
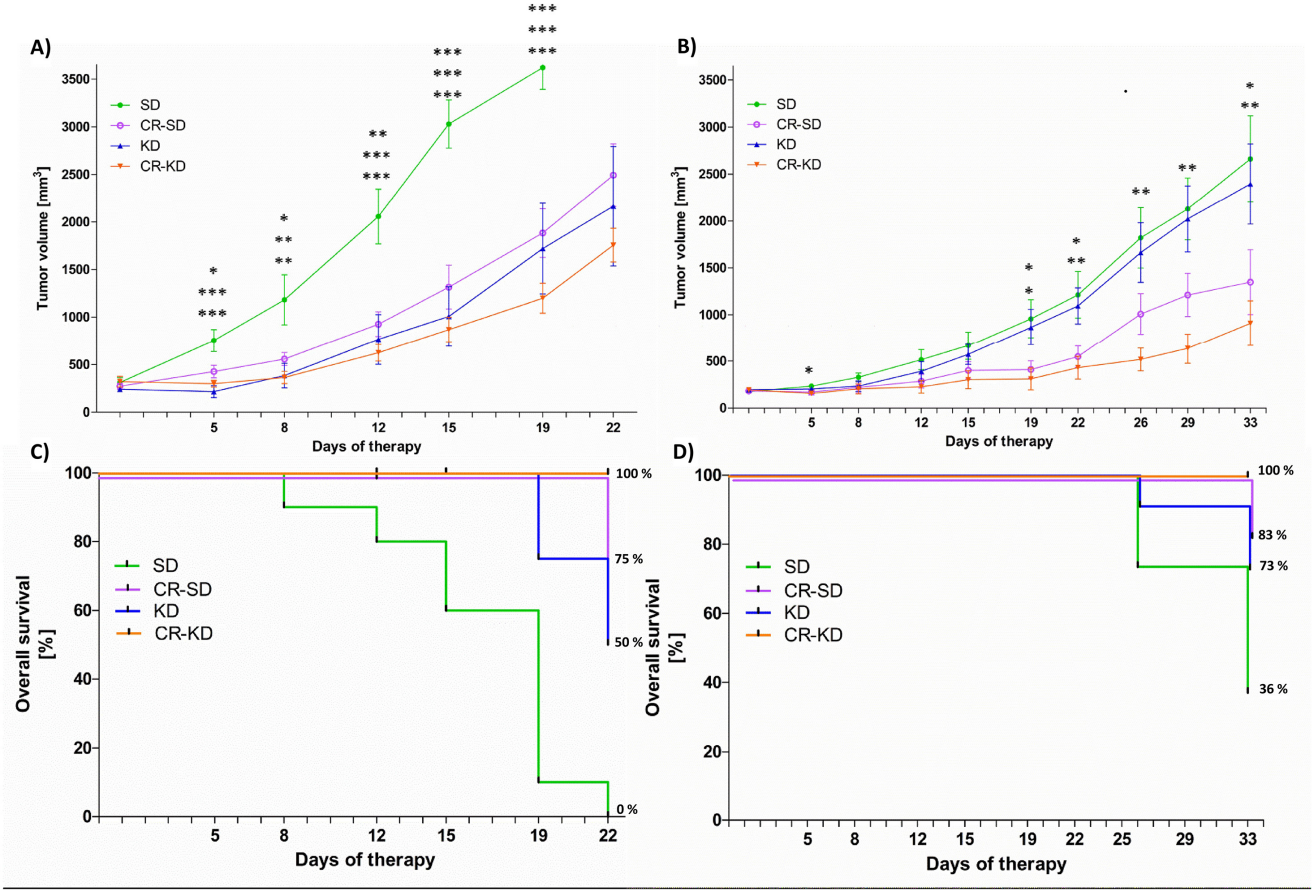
Ketogenic diet and calorie restriction reduce tumor growth and prolong survival in a NB xenograft model. After establishing tumors on the right flank of CD-1nu mice, the mice were randomized to diet groups as indicated. Tumor volume was measured twice weekly. A) For SH-SY5Y xenografts at day 19, the tumors of all diet groups showed significant growth inhibition compared to the SD group (CR-SD p = 0.001, KD p<0.001, CR-KD p<0.001). B) At day 33, SK-N-BE(2) tumor growth was significantly inhibited by CR (CR-SD p = 0.040, CR-KD p = 0.004). Inhibition of tumor growth was less pronounced in the KD group (p = 0.918). C) SH-SY5Y and D) SK-N-BE(2) show the results of Kaplan-Meier survival analysis of the corresponding treatment groups. Survival of mice with SH-SY5Y tumors at day 22 on SD was 0% compared to 75% on CR-SD (p<0.001), 50% on KD (p<0.001) and 100% on CR-KD (p<0.001). Survival of mice with SK-N-BE(2) xenografts at day 33 on SD was 36% compared to 83% on CR-SD (p = 0.017), 73% on KD (p = 0.09) and 100% on CR–KD (p<0.001). A, B) Data points for tumor growth curves represent mean values ± SEM of the corresponding diet group (n = 8–11). Statistics: ANOVA (p<0.05) followed by two-tailed Dunnett’s test correcting for multiple comparisons. C, D) Survival is expressed by the Kaplan–Meier method and differences between groups were determined in a univariate analysis with the log-rank test. Death is coded: tumor volume above 3000 mm^3^, tumor ulceration or impaired health condition. Diet groups are compared to the corresponding SD. * p≤0.05; ** p≤0.01; *** p≤0.001.

The effect of KD and/or CR on tumor growth was evaluated in xenograft models of two NB cell lines (SH-SY5Y and SK-N-BE(2)) and compared to SD (n = 8–11 per diet group, Fig 1). Whereas the SH-SY5Y cell line shows more benign characteristics and is sensitive to chemotherapy, the SK-N-BE(2) cell line is highly resistant to a wide range of chemotherapeutic agents. Moreover it displays markers of poor prognosis such as *NMYC* amplification, *TP53* mutation and chromosome 1p loss of heterozygosity [48]. Xenografts of the SH-SY5Y cell line experienced significant growth inhibition in all three dietary intervention groups (Fig 1A). On day 19, mean tumor volume in the SD group (3541 ± 219 mm^3^) was significantly increased compared to the CR-SD group (1884 ± 256 mm^3^, p = 0.001), the KD group (1721 ± 478 mm^3^, p<0.001) and the CR-KD group (1199 ± 158 mm^3^ p<0.001). Tumor volumes are given for day 19 because all except one of the tumors of the SD group reached the maximal volume of 3000 mm^3^ before day 22. Survival for mice on SD at day 22 was 0% compared to 75% on CR-SD (p<0.001), 50% on KD (p<0.001) and 100% on CR-KD (p<0.001) (Fig 1C). Tumor growth in the CR diet groups was significantly inhibited in the SK-N-BE(2) xenografts (CR-SD 1348 ± 345 mm^3^, p = 0.040, and CR-KD 909 ± 240 mm^3^ p = 0.004) when compared to the SD group (2661 ± 418 mm^3^). Tumor growth in the KD group was not significantly altered (2395 ± 426 mm^3^, p = 0.918) (Fig 1B). Survival of mice with SK-N-BE(2) xenografts on SD at day 33 was 36% compared to 83% on CR-SD (p = 0.017), 73% on KD (p = 0.09) and 100% on CR–KD (p<0.001) (Fig 1).

### Dietary intervention induces metabolic adaptations

Metabolic adaptation of mice to the different dietary interventions shows consistent patterns in the SH-SY5Y and SK-N-BE(2) (Table 1) therapy groups. CR induced a significant decrease in mean blood glucose levels when compared to SD (SH-SY5Y: CR-SD p = 0.002, CR-KD p = 0.002; SK-N-BE(2): CR-SD p<0.001, CR-KD p<0.001). No significant blood glucose change was detected in the KD groups (SH-SY5Y: p = 0.060; SK-N-BE(2): p = 0.092). Ketone bodies were increased in all intervention groups compared to SD, for SH-SY5Y significant only in the CR-KD group (p<0.001); for SK-N-BE(2) in all therapy groups (CR-SD p = 0.036, KD p<0.001, CR-KD p<0.001). Body weight change on the last day of therapy for CR-SD was significant only in the SH-SY5Y group (p = 0.01) and in both groups for CR-KD (SH-SY5Y: p<0.001; SK-N-BE(2): p = 0.01). Body weight loss in the CR groups was consistently below 20%.

**Table 1:** Changes in metabolic parameters of individual therapy groups.

Diet Group	blood glucose [mmol/l]	p^a^ vs SD	blood ketone^b^ [mmol/l]	p^a^ vs SD	BG/BK ratio^c^	body weight [g]	p^a^ vs SD
SD	7.9 ± 0.1	n.a.	0.5 ± 0.03	n.a.	15.80	23.15 ± 1.5	n.a.
CR-SD	6.4 ± 0.3	<0.001	0.7 ± 0.06	0.036	9.04	20.5 ± 0.6	0.108
KD	8.6 ± 0.1	0.092	0.9 ± 0.06	<0.001	9.56	25.21 ± 0.6	0.260
CR-KD	5.9 ± 0.2	<0.001	1.1 ± 0.08	<0.001	5.36	19.2 ± 0.6	0.01

^a^Statistics: ANOVA followed by two-tailed Dunnett´s test correcting for multiple comparisons. Diet groups are compared to the SD group. Data are given as mean ± SEM of the average metabolic parameter [blood glucose, blood ketone (^b^beta-hydroxybutyrate) or body weight].

^c^Blood glucose (BG, mmol/l) to blood ketone (BK, mmol/l) ratio, previously reported as Glucose Ketone Index [60]. Each group n = 11. n.a.: not applicable.

### Proliferation indices suggest G0 or early G1 arrest as a mechanism of reduced tumor growth

**Fig 2:**
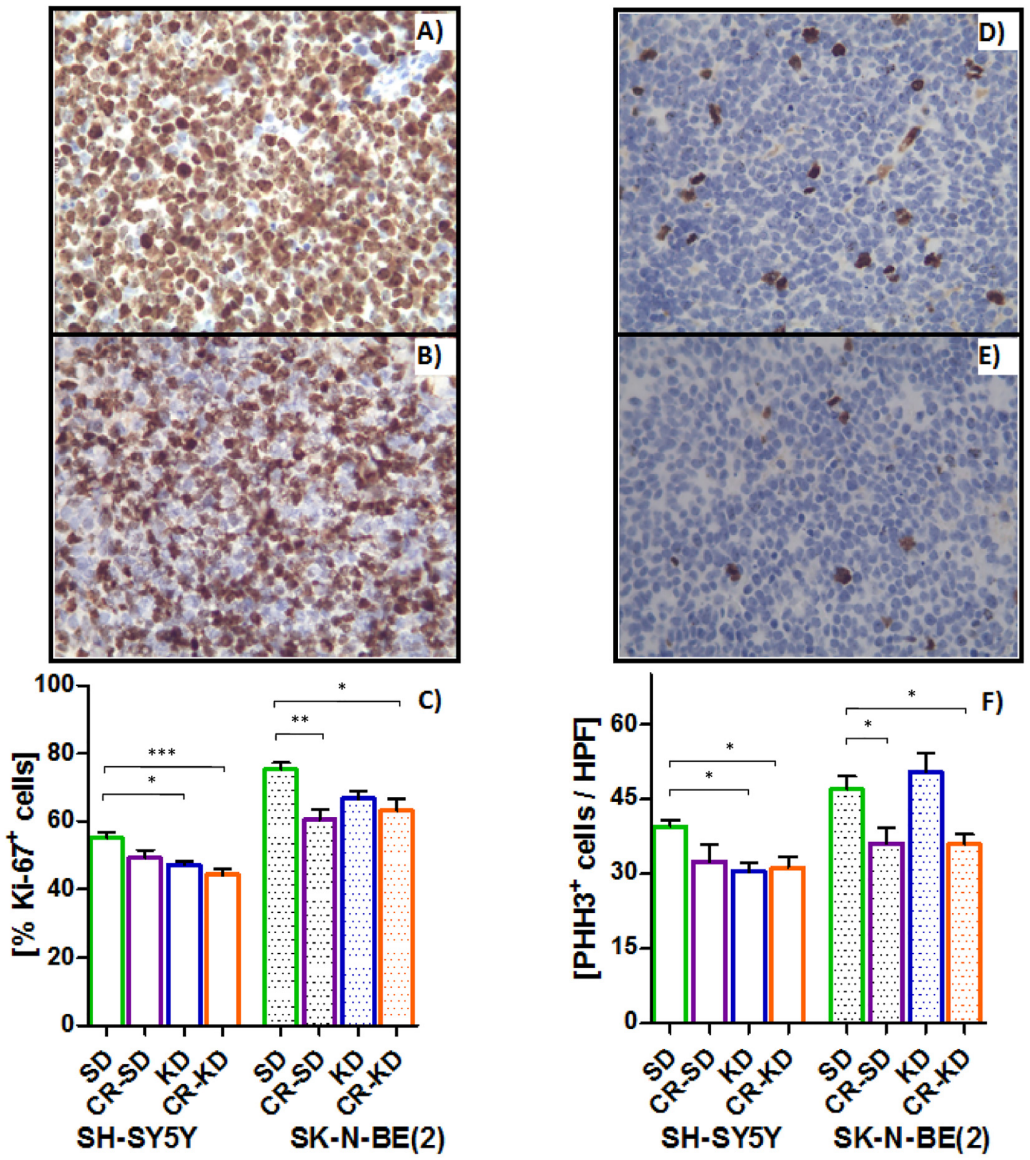
Proliferation indices Ki-67 (A-C) and PHH3 (D-F) suggest reduced proliferation by G0 or early G1 arrest. CR reduces proliferation in both cell lines whereas KD caused no reduction of proliferative activity in the SK-N-BE(2) xenograft group. A) shows a representative SD Ki-67 staining pattern in comparison to the CR-KD pattern (B) in SK-N-BE(2) tumor samples. For SH-SY5Y xenografts, PHH3 staining is depicted in D) and E) for SD and CR-KD treatment, respectively. Results are given as mean ± SEM. Statistics: ANOVA (p <0.05) followed by Dunnett’s test correcting for multiple comparisons. Diet groups are compared to the corresponding SD. * p≤0.05; ** p≤0.01; *** p≤0.001.

Proliferation indices Ki-67 and phospho-histone H3 (PHH3) were determined to elucidate if growth-inhibition in the distinct dietary intervention groups is a cause of the decreased proliferative activity. The measured reduction in the fraction of cycling cells is in line with the decrease of tumor growth in the corresponding diets (Fig 2). In the SH-SY5Y xenografts, the percentage of Ki67-positive staining significantly decreased from 55 ± 2% in the SD group to 47 ± 1% in the KD group (p = 0.015) and to 44 ± 2% in the CR-KD group (p = 0.001). In the SK-N-BE(2) xenografts, Ki67 staining was significantly decreased from 75 ± 2% in the SD group to 60 ± 3% in the CR-SD group (p<0.002) and to 63 ± 3% in the CR-KD group (p = 0.02) (Fig 2). The PHH3 results are congruent, depicting a significant decrease in positively stained nuclei from 39 ± 1/HPF (high power field) in the SD group to 30 ± 2/HPF in the KD group (p = 0.028) and 31 ± 2/HPF in the CR-KD group (p = 0.034) of the SH-SY5Y xenografts. In the SK-N-BE(2) xenografts, actively dividing cells were reduced from 47 ± 3/HPF in the SD group to 36 ± 3/HPF in the CR-SD group (p<0.05) and 35 ± 2/HPF in the CR-KD group (p<0.05).

### NB xenografts show no adaptation of mtDNA copy number or OXPHOS enzyme activities to dietary intervention

**Fig 3:**
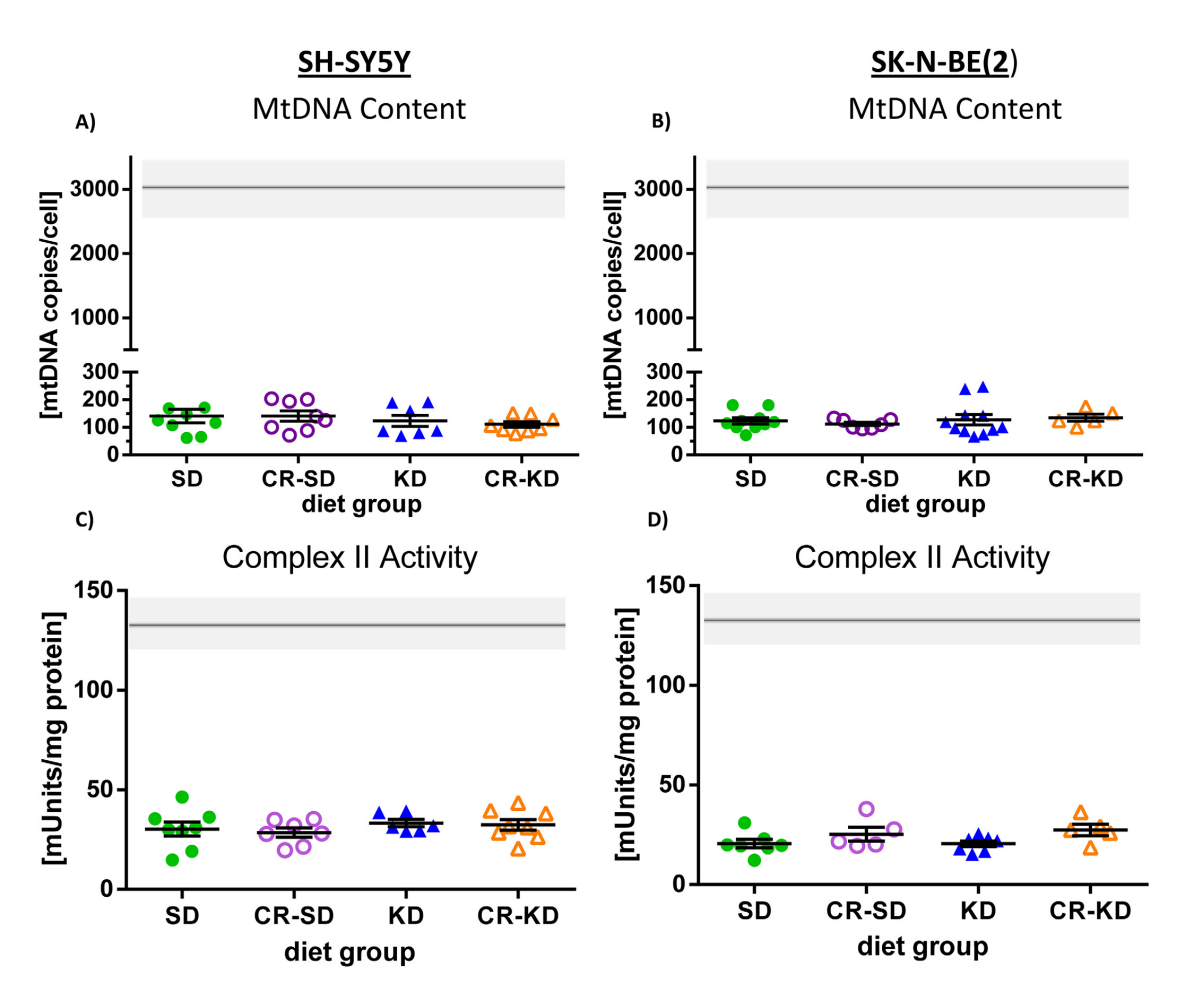
Evaluation of the adaptive response in mtDNA copy number and OXPHOS enzyme activities in NB xenografts to dietary intervention. Both cell types SH-SY5Y A) and SK-N-BE(2) C) show consistent low copies of the mitochondrial genome (mean <150). Panels B) SH-SY5Y and D) SK-N-BE(2) show mean complex II (succinate dehydrogenase) enzyme activities in xenografts of the different diet groups. Enzyme activities of all individual OXPHOS complexes are additionally given in the S1 and S2 Figs. No significant differences were detected between the different diet groups. For the SK-N-BE(2) xenografts, only representative samples were measured (n≥5). Statistics: ANOVA (p <0.05) followed by two-tailed Dunnett’s test correcting for multiple comparisons. Diet groups are compared to the corresponding SD group. The gray bars represent control values from kidney cortex tissue as reported previously [3].

NB tumors have more than 15-fold less mtDNA copies than normal human brain or kidney tissue (194 ± 22 [3] versus 3448 ± 524 [45] or 2960 ± 460 [3] respectively; mean ± SEM). Even when compared to human glioblastoma, a low-OXPHOS tumor entity, the amount of mtDNA copies is approximately 6-fold less (1194 ± 416 [45]; mean ± SEM). Under SD, mtDNA copies were 141 ± 24 (n = 9) in SH-SY5Y and 124 ± 11 (n = 10) in SK-N-BE(2) xenografts, matching with data from primary NB tumors. Dietary intervention did not influence mtDNA copies when compared to levels in the corresponding SD group (SH-SY5Y: CR-SD 141 ± 19 (n = 8), KD 124 ± 21 (n = 7), CR-KD 111 ± 10 (n = 8). SK-N-BE(2): CR-SD 113 ± 6 (n = 7), KD 128 ± 19 (n = 11), CR-KD 135 ± 13 (n = 5); Fig 3). No significant differences between *NMYC*-amplified (SK-N-BE(2)) and non-amplified (SH-SY5Y) xenografts were detected (p>0.05).

Low OXPHOS enzyme activities are compatible with *in vivo* data of NB tissue, supporting the used animal model [3]. As in primary tumor tissues, respiratory chain complex II showed the lowest levels and complex III activity is relatively stronger preserved. Especially in SH-SY5Y xenografts also complex IV activity was stronger preserved, when compared to primary neuroblastoma tissue. Complex II enzyme activity in all diet groups of the SH-SY5Y and the SK-N-BE(2) cell line-derived xenografts did not differ significantly (Fig 2, S1 and S2 Figs). Comprehensive enzymatic evaluation of all individual OXPHOS complexes (I-V) and citric acid synthase (CS, indicative of mitochondrial mass) showed no significant difference when compared to the activity of the corresponding SD group (p>0.05) (S1 and S2 Figs).

### OXPHOS complex western blot analysis and immunohistochemical staining support low enzymatic activities

**Fig 4:**
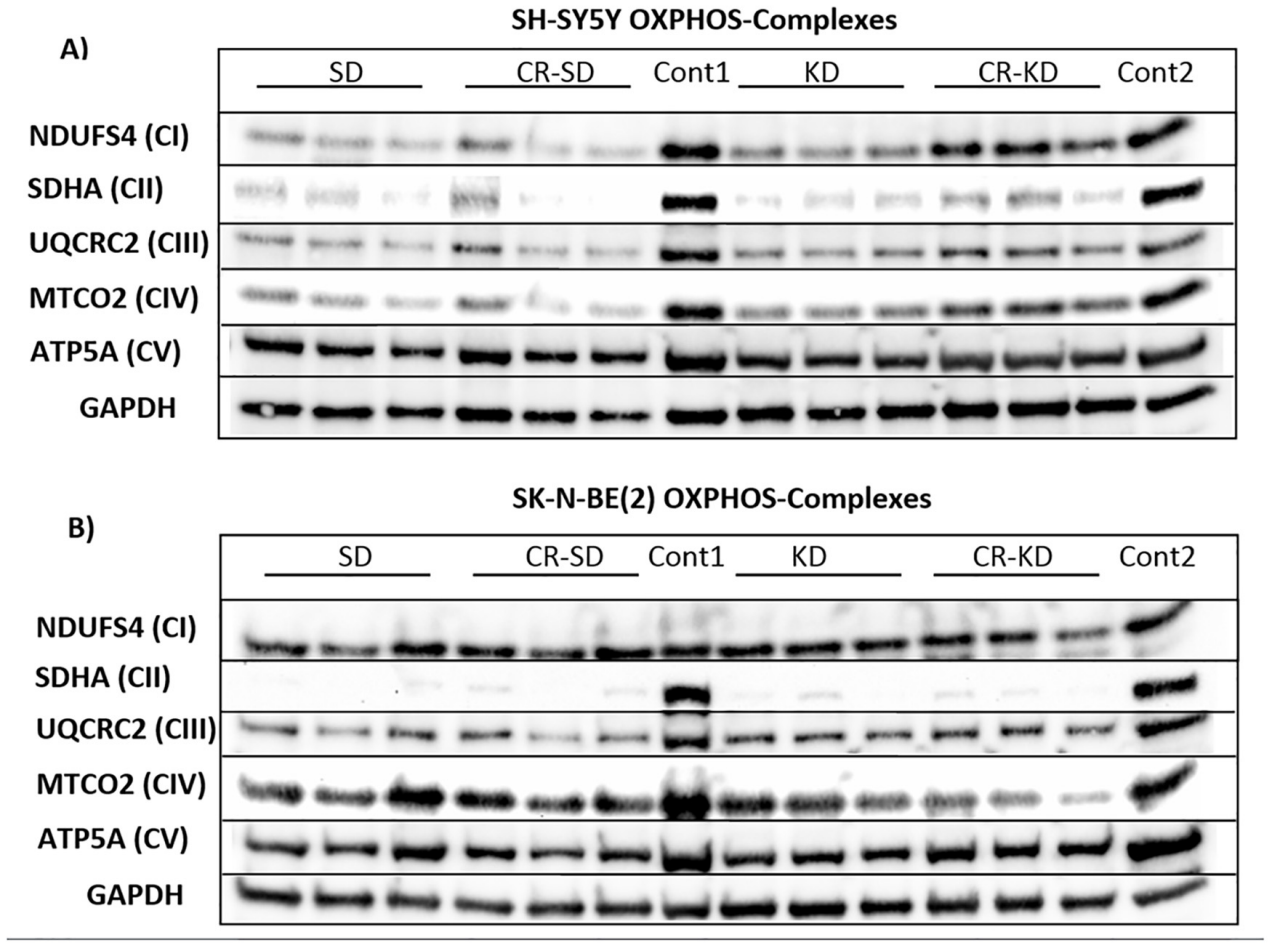
Western blot analysis of subunits of OXPHOS complexes (CI-CV) from SH-SY5Y (A) and SK-N-BE(2) (B) xenografts. Three samples from each diet group were loaded as indicated at the top (SD, CR-SD, KD and CR-KD). Representative subunits of CI-CV were probed as given on the left. Both cell types show consistent strong downregulation of CII, with the other complexes being relatively more preserved at the protein level. GAPDH is shown as loading control. As controls (cont1 and cont2), kidney cortex was used as described in [3]. Quantification of loading adjusted staining intensities is given in S3 Fig.

Three representative tumor samples of all diet groups were evaluated for complex I-V protein levels (Fig 4). The consistently low enzymatic activities with no significant changes in enzymatic levels are supported by the OXPHOS complex protein levels on western blot analysis. Quantification of loading adjusted staining intensities is given in S3 Fig. Xenografts of both cell types show very low abundance of CII. CI, CIII and CIV showed a tendency towards increased protein levels in the CR-KD group of the SH-SY5Y cell line. CV protein levels of the SH-SY5Y tumors and CI of the SK-N-BE(2) tumors, did not mirror the respective enzymatic activities. This might reflect a low enzyme activity that is not linked to the protein stability of the analyzed subunit.

**Fig 5:**
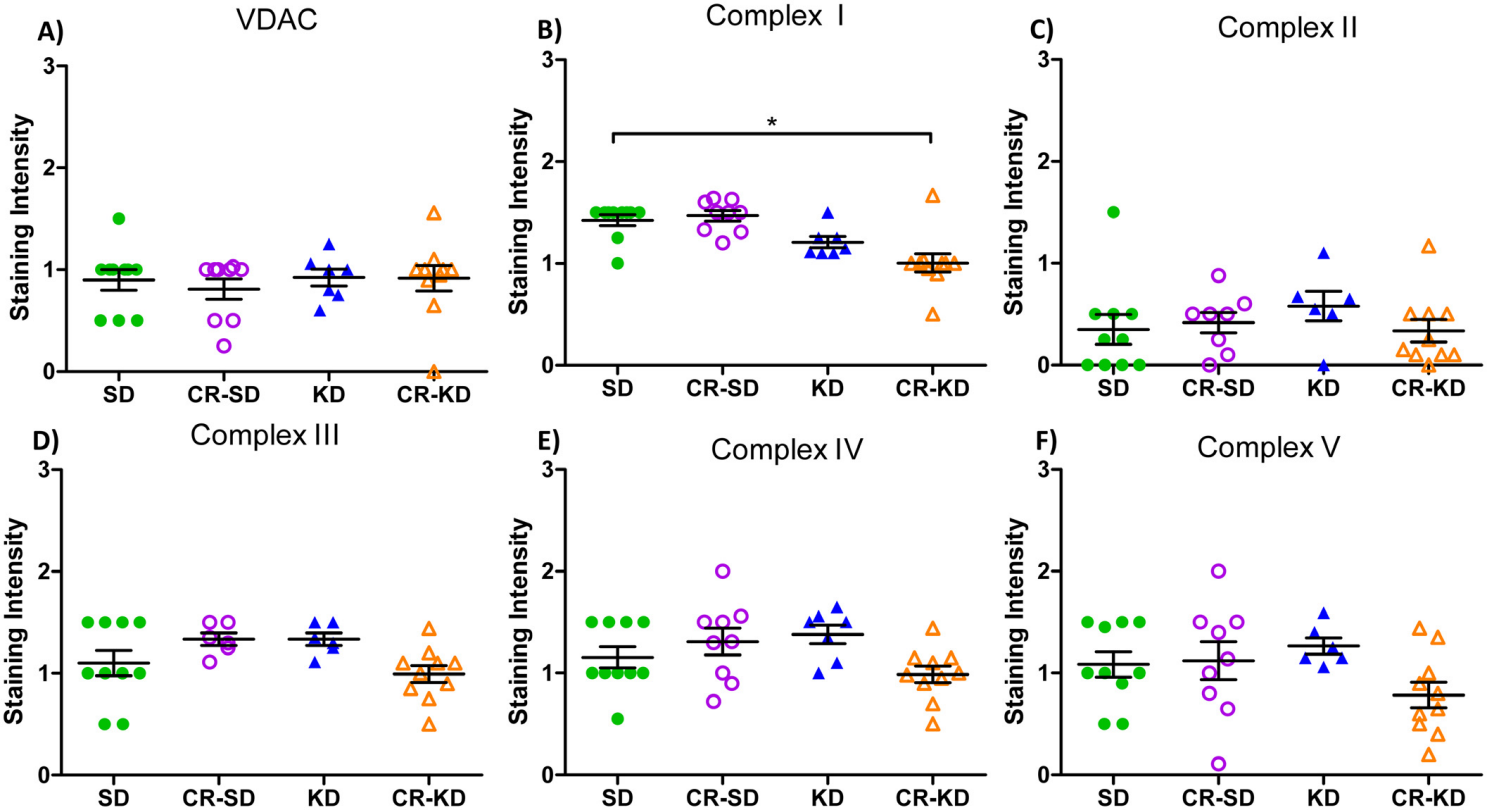
Immunohistochemical (IHC) staining of mitochondrial OXPHOS complexes I-V and VDAC in SH-SY5Y xenograft tumors. IHC staining of NB sections were scored on a scale from 0–3 as described in the methods section. The CR-KD group showed a significant decrease in complex I staining. All other evaluated parameters were unaffected by dietary changes. Voltage-dependent ion channel (VDAC) protein levels are used as a surrogate marker of mitochondrial mass. Statistics: ANOVA (p <0.05) followed by two-tailed Dunnett’s test correcting for multiple comparisons. Diet groups are compared to the corresponding SD group.

Immunohistochemical analysis of individual OXPHOS complexes (I-V) performed in the SH-SY5Y xenografts revealed a significant decrease in complex I staining in the CR-KD group when compared to the SD group (Fig 5; p<0.05). All other groups and complexes (II-V) were unaltered by dietary intervention.

### Succinyl-CoA:3-ketoacid coenzyme A transferase 1 (SCOT) protein levels are low in NB xenografts

Consistent with previous reports [47, 61, 62], SCOT, the rate-limiting enzyme in ketone body utilization, showed very low to non-detectable protein levels in western blot analysis of NB xenografts, whereas SCOT is prominent in control kidney tissue. SCOT levels did not change in response to the shift in nutrient supply induced by the different dietary interventions (S4 Fig).

## Discussion

To our knowledge, this is the first study demonstrating that targeting the metabolic phenotype of NB by dietary intervention can influence growth of this tumor entity. In the presented xenograft model KD and/or CR significantly reduced tumor growth and prolonged survival. The observed effects support the results of tumor models of the CNS, where CR-KD showed inhibitory effects on tumor growth in the pre-clinical setting [31, 40–42]. Applying KD without CR would reduce the burden for patients undergoing adjuvant dietary therapy, and support clinical feasibility. Further studies are needed to extend the presented data.

In line with the more pronounced resistance of the SK-N-BE(2) cell line to standard treatment, xenografts also proved to be more resistant to KD in our xenograft model. We hypothesize that there are factors besides OXPHOS capacity that influence the sensitivity towards dietary intervention as SK-N-BE(2) xenografts show a tendency towards lower enzymatic activities. An explanation that might contribute to this observation is the proposed influence of the NMYC protein on increasing glutamine uptake and utilization as an alternative substrate [63]. Being well aware of the limitations of pre-clinical studies, we regard the proof of concept and validation of tumor metabolic parameters as an important step to trigger further research in this area. Although the presented model utilizes an immune-compromised host and a rather non-physiologic tumor location, we specifically chose this model to reduce additional influences and allow extensive evaluation of the mitochondrial phenotype of tumor tissue. These methods are in line with those of other KD mouse models [27, 64–66].

Different mechanisms might contribute to the growth inhibitory potential of KD and/or CR on NB cancer cells observed in the presented model. A widely accepted theory is that the strong dependency on glycolysis for anabolic processes and energy production renders cancer cells susceptible to the decreased availability of glucose in hypovascularized tumor tissue [67–69]. In comparison with non malignant cells, cancer cells were shown to have reduced adaptability to the change in nutrient supply induced by KD and/or CR [31, 40–42]. Our data on the low SCOT expression in NB cells are in line with the reported inability of cancer cells to utilize ketone bodies as an energy source [61, 62]. Additionally ketone bodies might exert direct anti-proliferative effects in NB [47]. Results of KI67 and PHH3 staining suggest that reduced proliferation contributes to the observed tumor growth reduction in this model. As nutrient and energy depletion were shown to mediate cell cycle inhibition by LKB1-AMPK-pathway activation [70, 71] and mTOR-pathway inhibition [72, 73] these pathways constitute central targets for future investigations on the mechanisms of CR and/or KD. Besides reducing energy supply, glucose deprivation and KD was also correlated to thioredoxin redox system imbalance by reducing pentose phosphate pathway mediated NADPH production. This was linked to increased reactive oxygen species and toxicity in cancer cells [27, 74, 75]. Especially with regards to preclinical brain cancer models, also anti-angiogenic effects were reported [31, 32]. We are convinced that investigating the molecular mechanisms leading to growth inhibition of cancer cells in response to KD and/or CR will enhance our understanding of cancer cell biology and help to improve treatment strategies.

Deficiency in mitochondrial respiratory chain complex II activity is a well-described characteristic of familial paragangliomas and pheochromocytomas [76, 77]. Feichtinger et al. showed in 2010 that NB, another neural crest-derived tumor entity, shares this phenotype of distinctly low complex II activity. Although chromosomal region 1p36, where the complex II subunit-encoding *SDHB* gene maps, shows frequent loss of heterozygosity in NB, no mutation or epigenetic inactivation in any of the known complex II subunit-encoding genes has consistently been reported [3, 78, 79]. Additionally this low differentiated tumor entity shows a generalized low OXPHOS activity [3]. In the present study, we demonstrated that cell line-derived NB xenografts exhibit a similar OXPHOS phenotype as primary NB and might constitute a valuable resource for investigating the metabolic phenotype of NB. Complex II is the only subunit of the OXPHOS chain entirely encoded by the nuclear genome and is not dependent on cellular mtDNA content. The low complex II activity therefore implies a downregulation or deficiency of nuclear encoded OXPHOS subunits independent of the low mtDNA content. Complex II is also the only enzyme directly linking the citric acid cycle with OXPHOS. Future studies with this model might facilitate understanding of the regulation of mitochondrial OXPHOS complex activity and expression in cancer cells.

The more generalized low mitochondrial OXPHOS activity observed in NB corresponds to the low mtDNA copy number. This state of low mtDNA content is shared by other predominantly glycolytic cell types such as neural stem cells and glioblastoma multiforme cancer cells and is suggested to be an important feature of low differentiation status [80, 81]. Compared to glioblastoma, however, downregulation of mtDNA in NB is even more pronounced (approximately 6-fold) [3, 45]. Upon inducing differentiation, neural stem cells were shown to undergo a concerted expansion of mtDNA copy number, resulting in an increase in respiratory capacity. In comparison, glioblastoma cells showed a significantly reduced capacity to do so [80]. These results imply a direct connection of OXPHOS activity and mtDNA content to differentiation status and/or malignant phenotype. Besides their crucial role in cellular metabolism, mitochondria acceptedly serve central functions in the induction of apoptosis under physiological conditions and in cancer cells [82]. Most intriguingly the execution of this function was shown to be dependent on preserved OXPHOS in different model systems [83–85]. Both, depleting mtDNA and introducing mtDNA mutations via a cybrid-cell approach, were capable of mediating resistance to the induction of mitochondria-mediated apoptosis [83–86]. MtDNA content might therefore constitute a link between the low differentiation status and resistance to apoptosis. We showed in our xenograft model that NB tumors cannot adapt mitochondrial energy metabolism to the changes in nutrient supply induced with dietary intervention. This trait is supported by observations of sustained low OXPHOS activity, mtDNA copy number evaluation and protein quantification, and was independent of *NMYC* status or chromosome 1p36 deletion. The molecular mechanisms underlying this low mtDNA phenotype of NB are still to be determined. In view of the pseudo-hypoxic state that is discussed for complex II-deficient cells, one might speculate that this phenotype causes a secondary downregulation of cellular mtDNA replication [87]. Interestingly *in vitro* experiments utilizing decanoic acid (C10), a medium chain fatty acid, have recently provided evidence for peroxisome proliferator-activated receptor γ induced mitochondrial biogenesis in SH-SY5Y cells [88]. Since we did not observe this effect in our long chain fatty acid based KD it might be of interest to further evaluate the effects of MCT-based ketogenic diets on NB tumors.

One of the main advantages of KD over developing metabolism-targeting compounds is its ready accessibility for clinical evaluation and the lengthy past experience with clinical application in epilepsy [34]. Especially in the pediatric population, KD may represent a facile, at-hand, and tolerable adjunctive treatment strategy. In adult advanced-stage cancer patients, KD showed no major side effects [28, 30]. More extensive pre-clinical evaluation and strategies to introduce KD to NB cancer patients under controlled conditions will be mandatory to allow possible clinical application.

## Conclusions

Targeting the metabolism of cancer cells is an emerging strategy with the potential to improve currently insufficient treatment modalities. To do so we need to validate tumor models and improve characterization of native tissue with regard to cell metabolism. Here we showed that cell line-derived NB xenografts match the OXPHOS parameters of patient-derived tumor tissue and are therefore a valuable tool for preclinical studies. Using this model, we demonstrated that CR and/or KD reduce NB tumor growth and that tumor cells do not express adaptive mechanisms regarding the OXPHOS system. Therefore, we propose that KD and/or CR should be further evaluated as a possible adjuvant therapy in patients undergoing treatment for NB.

## Supporting Information

S1 Fig Activities of mitochondrial OXPHOS complexes I-V of SH-SY5Y xenograft tumors. (PDF)

S2 Fig Activities of mitochondrial OXPHOS complexes I-V of SK-N-BE(2) xenograft tumors. (PDF)

S3 Fig Staining intensities of mitochondrial OXPHOS complexes I-V relative to kidney cortex (Fig 4). (PDF)

S4 Fig Western blot analysis of SCOT expression in NB xenografts. (PDF)

S1 Table Ingredient list of the two diets. (PDF)

S2 Table MtDNA copy number primer list. (PDF)

## References

[1] Brodeur GM. Neuroblastoma: biological insights into a clinical enigma. Nat Rev Cancer. 2003;3(3):203–16. 10.1038/nrc1014 . 12612655

[2] Park JR, Bagatell R, London WB, Maris JM, Cohn SL, Mattay KM, et al Children's Oncology Group's 2013 blueprint for research: neuroblastoma. Pediatric blood & cancer. 2013;60(6):985–93. 10.1002/pbc.24433 . 23255319

[3] Feichtinger RG, Zimmermann F, Mayr JA, Neureiter D, Hauser-Kronberger C, Schilling FH, et al Low aerobic mitochondrial energy metabolism in poorly- or undifferentiated neuroblastoma. BMC cancer. 2010;10:149 Epub 2010/04/20. 10.1186/1471-2407-10-149 20398431 PMC2861660

[4] Schneider L, Giordano S, Zelickson BR, M SJ, G AB, Ouyang X, et al Differentiation of SH-SY5Y cells to a neuronal phenotype changes cellular bioenergetics and the response to oxidative stress. Free radical biology & medicine. 2011;51(11):2007–17. 10.1016/j.freeradbiomed.2011.08.030 21945098 PMC3208787

[5] Chiang MC, Cheng YC, Chen HM, Liang YJ, Yen CH. Rosiglitazone promotes neurite outgrowth and mitochondrial function in N2A cells via PPARgamma pathway. Mitochondrion. 2013;14C:7–17. 10.1016/j.mito.2013.12.003 . 24370585

[6] Xun Z, Lee DY, Lim J, Canaria CA, Barnebey A, Yanonne SM, et al Retinoic acid-induced differentiation increases the rate of oxygen consumption and enhances the spare respiratory capacity of mitochondria in SH-SY5Y cells. Mechanisms of ageing and development. 2012;133(4):176–85. 10.1016/j.mad.2012.01.008 22336883 PMC3357086

[7] Hanahan D, Weinberg RA. Hallmarks of cancer: the next generation. Cell. 2011;144(5):646–74. 10.1016/j.cell.2011.02.013 . 21376230

[8] Vander Heiden MG, Cantley LC, Thompson CB. Understanding the Warburg effect: the metabolic requirements of cell proliferation. Science. 2009;324(5930):1029–33. Epub 2009/05/23. doi: 324/5930/1029 [pii] 10.1126/science.1160809 19460998 PMC2849637

[9] Kim JW, Dang CV. Cancer's molecular sweet tooth and the Warburg effect. Cancer research. 2006;66(18):8927–30. Epub 2006/09/20. doi: 66/18/8927 [pii] 10.1158/0008-5472.CAN-06-1501 . 16982728

[10] Warburg O. Über den Stoffwechsel der Carcinomzelle. Die Naturwissenschaften. 1924;50:1131–7.

[11] Meierhofer D, Mayr JA, Foetschl U, Berger A, Fink K, Schmeller N, et al Decrease of mitochondrial DNA content and energy metabolism in renal cell carcinoma. Carcinogenesis. 2004;25(6):1005–10. . 14764459 10.1093/carcin/bgh104

[12] Feichtinger RG, Neureiter D, Mayr JA, Zimmermann FA, Berthold F, Jones N, et al Loss of mitochondria in ganglioneuromas. Front Biosci (Elite Ed). 2011;3:179–86. Epub 2011/01/05. . 21196296 10.2741/e231

[13] Feichtinger RG, Neureiter D, Royer-Pokora B, Mayr JA, Zimmermann FA, Jones N, et al Heterogeneity of mitochondrial energy metabolism in classical triphasic Wilms' tumor. Front Biosci (Elite Ed). 2011;3:187–93. Epub 2011/01/05. . 21196297 10.2741/e232

[14] Feichtinger RG, Zimmermann FA, Mayr JA, Neureiter D, Ratschek M, Jones N, et al Alterations of respiratory chain complexes in sporadic pheochromocytoma. Front Biosci (Elite Ed). 2011;3:194–200. Epub 2011/01/05. . 21196298 10.2741/e233

[15] Mayr JA, Meierhofer D, Zimmermann F, Feichtinger R, Kogler C, Ratschek M, et al Loss of complex I due to mitochondrial DNA mutations in renal oncocytoma. Clinical cancer research: an official journal of the American Association for Cancer Research. 2008;14(8):2270–5. 10.1158/1078-0432.CCR-07-4131 18413815

[16] Zimmermann FA, Mayr JA, Feichtinger R, Neureiter D, Lechner R, Koegler C, et al Respiratory chain complex I is a mitochondrial tumor suppressor of oncocytic tumors. Front Biosci (Elite Ed). 2011;3:315–25. Epub 2011/01/05. . 21196312 10.2741/e247

[17] Zimmermann FA, Mayr JA, Neureiter D, Feichtinger R, Alinger B, Jones ND, et al Lack of complex I is associated with oncocytic thyroid tumours. British journal of cancer. 2009;100(9):1434–7. 10.1038/sj.bjc.6605028 19352385 PMC2694433

[18] Lopez-Rios F, Sanchez-Arago M, Garcia-Garcia E, Ortega AD, Berrendero JR, Pozo-Rodriguez F, et al Loss of the mitochondrial bioenergetic capacity underlies the glucose avidity of carcinomas. Cancer research. 2007;67(19):9013–7. 10.1158/0008-5472.CAN-07-1678 . 17909002

[19] Gambhir SS. Molecular imaging of cancer with positron emission tomography. Nat Rev Cancer. 2002;2(9):683–93. 10.1038/nrc882 . 12209157

[20] Vander Heiden MG. Targeting cancer metabolism: a therapeutic window opens. Nature reviews Drug discovery. 2011;10(9):671–84. 10.1038/nrd3504 . 21878982

[21] Zhang D, Li J, Wang F, Hu J, Wang S, Sun Y. 2-Deoxy-D-glucose targeting of glucose metabolism in cancer cells as a potential therapy. Cancer letters. 2014;355(2):176–83. 10.1016/j.canlet.2014.09.003 . 25218591

[22] Raez LE, Papadopoulos K, Ricart AD, Chiorean EG, Dipaola RS, Stein MN, et al A phase I dose-escalation trial of 2-deoxy-D-glucose alone or combined with docetaxel in patients with advanced solid tumors. Cancer Chemother Pharmacol. 2013;71(2):523–30. Epub 2012/12/12. 10.1007/s00280-012-2045-1 . 23228990

[23] Jones NP, Schulze A. Targeting cancer metabolism—aiming at a tumour's sweet-spot. Drug discovery today. 2012;17(5–6):232–41. 10.1016/j.drudis.2011.12.017 . 22207221

[24] Butler EB, Zhao Y, Munoz-Pinedo C, Lu J, Tan M. Stalling the engine of resistance: targeting cancer metabolism to overcome therapeutic resistance. Cancer research. 2013;73(9):2709–17. 10.1158/0008-5472.CAN-12-3009 23610447 PMC3644012

[25] Tennant DA, Duran RV, Gottlieb E. Targeting metabolic transformation for cancer therapy. Nat Rev Cancer. 2010;10(4):267–77. Epub 2010/03/20. doi: nrc2817 [pii] 10.1038/nrc2817 . 20300106

[26] Lv M, Zhu X, Wang H, Wang F, Guan W. Roles of caloric restriction, ketogenic diet and intermittent fasting during initiation, progression and metastasis of cancer in animal models: a systematic review and meta-analysis. PloS one. 2014;9(12):e115147 Epub 2014/12/17. 10.1371/journal.pone.0115147 ; PubMed Central PMCID: PMCPmc4263749. 25502434 PMC4263749

[27] Allen BG, Bhatia SK, Buatti JM, Brandt KE, Lindholm KE, Button AM, et al Ketogenic diets enhance oxidative stress and radio-chemo-therapy responses in lung cancer xenografts. Clinical cancer research: an official journal of the American Association for Cancer Research. 2013;19(14):3905–13. 10.1158/1078-0432.CCR-12-0287 23743570 PMC3954599

[28] Rieger J, Bahr O, Maurer GD, Hattingen E, Franz K, Brucker D, et al ERGO: A pilot study of ketogenic diet in recurrent glioblastoma. International journal of oncology. 2014;44(6):1843–52. Epub 2014/04/15. 10.3892/ijo.2014.2382 . 24728273 PMC4063533

[29] Chu-Shore CJ, Thiele EA. Tumor growth in patients with tuberous sclerosis complex on the ketogenic diet. Brain Dev. 2010;32(4):318–22. Epub 2009/05/16. doi: S0387-7604(09)00136-3 [pii] 10.1016/j.braindev.2009.04.009 . 19443154

[30] Schmidt M, Pfetzer N, Schwab M, Strauss I, Kammerer U. Effects of a ketogenic diet on the quality of life in 16 patients with advanced cancer: A pilot trial. Nutrition & metabolism. 2011;8(1):54 Epub 2011/07/29. 10.1186/1743-7075-8-54 21794124 PMC3157418

[31] Mukherjee P, Abate LE, Seyfried TN. Antiangiogenic and proapoptotic effects of dietary restriction on experimental mouse and human brain tumors. Clinical cancer research: an official journal of the American Association for Cancer Research. 2004;10(16):5622–9. Epub 2004/08/26. doi: 10.1158/1078-0432.CCR-04-0308 10/16/5622 [pii]. . 15328205 10.1158/1078-0432.CCR-04-0308

[32] Mukherjee P, El-Abbadi MM, Kasperzyk JL, Ranes MK, Seyfried TN. Dietary restriction reduces angiogenesis and growth in an orthotopic mouse brain tumour model. British journal of cancer. 2002;86(10):1615–21. Epub 2002/06/27. 10.1038/sj.bjc.6600298 12085212 PMC2746602

[33] Seyfried TN, Mukherjee P. Targeting energy metabolism in brain cancer: review and hypothesis. Nutrition & metabolism. 2005;2:30. Epub 2005/10/26. doi: 1743-7075-2-30 [pii] 10.1186/1743-7075-2-30 16242042 PMC1276814

[34] Freeman JM, Kossoff EH, Hartman AL. The ketogenic diet: one decade later. Pediatrics. 2007;119(3):535–43. 10.1542/peds.2006-2447 . 17332207

[35] Payne NE, Cross JH, Sander JW, Sisodiya SM. The ketogenic and related diets in adolescents and adults—a review. Epilepsia. 2011;52(11):1941–8. 10.1111/j.1528-1167.2011.03287.x . 22004525

[36] Huffman J, Kossoff EH. State of the ketogenic diet(s) in epilepsy. Current neurology and neuroscience reports. 2006;6(4):332–40. . 16822355 10.1007/s11910-006-0027-6

[37] Hartman AL, Vining EP. Clinical aspects of the ketogenic diet. Epilepsia. 2007;48(1):31–42. 10.1111/j.1528-1167.2007.00914.x . 17241206

[38] Paoli A, Rubini A, Volek JS, Grimaldi KA. Beyond weight loss: a review of the therapeutic uses of very-low-carbohydrate (ketogenic) diets. European journal of clinical nutrition. 2013;67(8):789–96. 10.1038/ejcn.2013.116 23801097 PMC3826507

[39] Bough KJ, Wetherington J, Hassel B, Pare JF, Gawryluk JW, Greene JG, et al Mitochondrial biogenesis in the anticonvulsant mechanism of the ketogenic diet. Annals of neurology. 2006;60(2):223–35. 10.1002/ana.20899 . 16807920

[40] Seyfried TN, Shelton LM, Mukherjee P. Does the existing standard of care increase glioblastoma energy metabolism? The lancet oncology. 2010;11(9):811–3. Epub 2010/07/17. doi: S1470-2045(10)70166-2 [pii] 10.1016/S1470-2045(10)70166-2 . 20634134

[41] Seyfried TN, Kiebish MA, Marsh J, Shelton LM, Huysentruyt LC, Mukherjee P. Metabolic management of brain cancer. Biochimica et biophysica acta. 2011;1807(6):577–94. Epub 2010/09/02. doi: S0005-2728(10)00685-7 [pii] 10.1016/j.bbabio.2010.08.009 . 20804725

[42] Maroon J, Bost J, Amos A, Zuccoli G. Restricted calorie ketogenic diet for the treatment of glioblastoma multiforme. Journal of child neurology. 2013;28(8):1002–8. 10.1177/0883073813488670 . 23670248

[43] Zuccoli G, Marcello N, Pisanello A, Servadei F, Vaccaro S, Mukherjee P, et al Metabolic management of glioblastoma multiforme using standard therapy together with a restricted ketogenic diet: Case Report. Nutrition & metabolism. 2010;7:33 Epub 2010/04/24. 10.1186/1743-7075-7-33 20412570 PMC2874558

[44] Nebeling LC, Miraldi F, Shurin SB, Lerner E. Effects of a ketogenic diet on tumor metabolism and nutritional status in pediatric oncology patients: two case reports. J Am Coll Nutr. 1995;14(2):202–8. Epub 1995/04/01. . 7790697 10.1080/07315724.1995.10718495

[45] Gunther Feichtinger R, Weis S, Adalbert Mayr J, Zimmermann F, Geilberger R, Sperl W, et al Alterations of oxidative phosphorylation complexes in astrocytomas. Glia. 2014 Epub 2014/01/22. 10.1002/glia.22621 . 24446254

[46] Roeder LM, Poduslo SE, Tildon JT. Utilization of ketone bodies and glucose by established neural cell lines. J Neurosci Res. 1982;8(4):671–82. 10.1002/jnr.490080412 . 7161845

[47] Skinner R, Trujillo A, Ma X, Beierle EA. Ketone bodies inhibit the viability of human neuroblastoma cells. Journal of pediatric surgery. 2009;44(1):212–6; discussion 6. 10.1016/j.jpedsurg.2008.10.042 19159745

[48] Li Z, Yan S, Attayan N, Ramalingam S, Thiele CJ. Combination of an allosteric Akt Inhibitor MK-2206 with etoposide or rapamycin enhances the antitumor growth effect in neuroblastoma. Clinical cancer research: an official journal of the American Association for Cancer Research. 2012;18(13):3603–15. Epub 2012/05/03. 1078-0432.CCR-11-3321 [pii] 10.1158/1078-0432.CCR-11-3321 . 22550167 PMC6693338

[49] Laffel L. Ketone bodies: a review of physiology, pathophysiology and application of monitoring to diabetes. Diabetes/metabolism research and reviews. 1999;15(6):412–26. Epub 2000/01/15. . 10634967 10.1002/(sici)1520-7560(199911/12)15:6<412::aid-dmrr72>3.0.co;2-8

[50] Kristensen GB, Christensen NG, Thue G, Sandberg S. Between-lot variation in external quality assessment of glucose: clinical importance and effect on participant performance evaluation. Clin Chem. 2005;51(9):1632–6. Epub 2005/08/27. 10.1373/clinchem.2005.049080 . 16120948

[51] Kimberly MM, Vesper HW, Caudill SP, Ethridge SF, Archibold E, Porter KH, et al Variability among five over-the-counter blood glucose monitors. Clinica chimica acta; international journal of clinical chemistry. 2006;364(1–2):292–7. Epub 2005/09/07. 10.1016/j.cca.2005.07.027 . 16143321

[52] Mayr JA, Zimmermann FA, Fauth C, Bergheim C, Meierhofer D, Radmayr D, et al Lipoic acid synthetase deficiency causes neonatal-onset epilepsy, defective mitochondrial energy metabolism, and glycine elevation. Am J Hum Genet. 2011;89(6):792–7. Epub 2011/12/14. doi: S0002-9297(11)00489-7 [pii] 10.1016/j.ajhg.2011.11.011 22152680 PMC3234378

[53] Pon LA, Schon EA. Mitochondria. 2nd ed Amsterdam; Boston: Academic Press; 2007 xxiv, 920 p. p.

[54] Berger A, Mayr JA, Meierhofer D, Fotschl U, Bittner R, Budka H, et al Severe depletion of mitochondrial DNA in spinal muscular atrophy. Acta Neuropathol. 2003;105(3):245–51. . 12557011 10.1007/s00401-002-0638-1

[55] Holloszy JO, Oscai LB, Don IJ, Mole PA. Mitochondrial citric acid cycle and related enzymes: adaptive response to exercise. Biochem Biophys Res Commun. 1970;40(6):1368–73. Epub 1970/09/30. doi: 0006-291X(70)90017-3 [pii]. . 4327015 10.1016/0006-291x(70)90017-3

[56] Srere PA. Citrate synthase. Methods in enzymology. 1969;13:3–5. .4329195

[57] Rustin P, Chretien D, Bourgeron T, Gerard B, Rotig A, Saudubray JM, et al Biochemical and molecular investigations in respiratory chain deficiencies. Clinica chimica acta; international journal of clinical chemistry. 1994;228(1):35–51. . 7955428 10.1016/0009-8981(94)90055-8

[58] Kollberg G, Darin N, Benan K, Moslemi AR, Lindal S, Tulinius M, et al A novel homozygous RRM2B missense mutation in association with severe mtDNA depletion. Neuromuscul Disord. 2009;19(2):147–50. Epub 2009/01/14. doi: S0960-8966(08)00707-4 [pii] 10.1016/j.nmd.2008.11.014 . 19138848

[59] Acham-Roschitz B, Plecko B, Lindbichler F, Bittner R, Mache CJ, Sperl W, et al A novel mutation of the RRM2B gene in an infant with early fatal encephalomyopathy, central hypomyelination, and tubulopathy. Mol Genet Metab. 2009;98(3):300–4. 10.1016/j.ymgme.2009.06.012 19616983

[60] Meidenbauer JJ, Mukherjee P, Seyfried TN. The glucose ketone index calculator: a simple tool to monitor therapeutic efficacy for metabolic management of brain cancer. Nutrition & metabolism. 2015;12:12 10.1186/s12986-015-0009-2 25798181 PMC4367849

[61] Tisdale MJ, Brennan RA. Loss of acetoacetate coenzyme A transferase activity in tumours of peripheral tissues. British journal of cancer. 1983;47(2):293–7. Epub 1983/02/01. 6130780 10.1038/bjc.1983.38 PMC2011283

[62] Chang HT, Olson LK, Schwartz KA. Ketolytic and glycolytic enzymatic expression profiles in malignant gliomas: implication for ketogenic diet therapy. Nutrition & metabolism. 2013;10(1):47. Epub 2013/07/09. doi: 1743-7075-10-47 [pii] 10.1186/1743-7075-10-47 23829383 PMC3707813

[63] Qing G, Li B, Vu A, Skuli N, Walton ZE, Liu X, et al ATF4 regulates MYC-mediated neuroblastoma cell death upon glutamine deprivation. Cancer cell. 2012;22(5):631–44. 10.1016/j.ccr.2012.09.021 23153536 PMC3510660

[64] Venkateswaran V, Haddad AQ, Fleshner NE, Fan R, Sugar LM, Nam R, et al Association of diet-induced hyperinsulinemia with accelerated growth of prostate cancer (LNCaP) xenografts. J Natl Cancer Inst. 2007;99(23):1793–800. 10.1093/jnci/djm231 . 18042933

[65] Otto C, Kaemmerer U, Illert B, Muehling B, Pfetzer N, Wittig R, et al Growth of human gastric cancer cells in nude mice is delayed by a ketogenic diet supplemented with omega-3 fatty acids and medium-chain triglycerides. BMC cancer. 2008;8:122 10.1186/1471-2407-8-122 18447912 PMC2408928

[66] Freedland SJ, Mavropoulos J, Wang A, Darshan M, Demark-Wahnefried W, Aronson WJ, et al Carbohydrate restriction, prostate cancer growth, and the insulin-like growth factor axis. The Prostate. 2008;68(1):11–9. 10.1002/pros.20683 17999389 PMC3959866

[67] Seyfried TN, Sanderson TM, El-Abbadi MM, McGowan R, Mukherjee P. Role of glucose and ketone bodies in the metabolic control of experimental brain cancer. British journal of cancer. 2003;89(7):1375–82. Epub 2003/10/02. 10.1038/sj.bjc.6601269 14520474 PMC2394295

[68] Shim H, Chun YS, Lewis BC, Dang CV. A unique glucose-dependent apoptotic pathway induced by c-Myc. Proceedings of the National Academy of Sciences of the United States of America. 1998;95(4):1511–6. 9465046 10.1073/pnas.95.4.1511 PMC19067

[69] Dang CV, Hamaker M, Sun P, Le A, Gao P. Therapeutic targeting of cancer cell metabolism. J Mol Med (Berl). 2011;89(3):205–12. 10.1007/s00109-011-0730-x 21301795 PMC3345191

[70] Liang J, Shao SH, Xu ZX, Hennessy B, Ding Z, Larrea M, et al The energy sensing LKB1-AMPK pathway regulates p27(kip1) phosphorylation mediating the decision to enter autophagy or apoptosis. Nature cell biology. 2007;9(2):218–24. Epub 2007/01/24. 10.1038/ncb1537 . 17237771

[71] Hardie DG. AMP-activated protein kinase: an energy sensor that regulates all aspects of cell function. Genes & development. 2011;25(18):1895–908. Epub 2011/09/23. 10.1101/gad.17420111 21937710 PMC3185962

[72] Barbet NC, Schneider U, Helliwell SB, Stansfield I, Tuite MF, Hall MN. TOR controls translation initiation and early G1 progression in yeast. Molecular biology of the cell. 1996;7(1):25–42. Epub 1996/01/01. 8741837 10.1091/mbc.7.1.25 PMC278610

[73] Fingar DC, Richardson CJ, Tee AR, Cheatham L, Tsou C, Blenis J. mTOR controls cell cycle progression through its cell growth effectors S6K1 and 4E-BP1/eukaryotic translation initiation factor 4E. Molecular and cellular biology. 2004;24(1):200–16. Epub 2003/12/16. 14673156 10.1128/MCB.24.1.200-216.2004 PMC303352

[74] Aykin-Burns N, Ahmad IM, Zhu Y, Oberley LW, Spitz DR. Increased levels of superoxide and H2O2 mediate the differential susceptibility of cancer cells versus normal cells to glucose deprivation. The Biochemical journal. 2009;418(1):29–37. 10.1042/BJ20081258 18937644 PMC2678564

[75] Ahmad IM, Aykin-Burns N, Sim JE, Walsh SA, Higashikubo R, Buettner GR, et al Mitochondrial O2*- and H2O2 mediate glucose deprivation-induced stress in human cancer cells. J Biol Chem. 2005;280(6):4254–63. 10.1074/jbc.M411662200 . 15561720

[76] Baysal BE, Ferrell RE, Willett-Brozick JE, Lawrence EC, Myssiorek D, Bosch A, et al Mutations in SDHD, a mitochondrial complex II gene, in hereditary paraganglioma. Science. 2000;287(5454):848–51. . 10657297 10.1126/science.287.5454.848

[77] Amar L, Bertherat J, Baudin E, Ajzenberg C, Bressac-de Paillerets B, Chabre O, et al Genetic testing in pheochromocytoma or functional paraganglioma. J Clin Oncol. 2005;23(34):8812–8. Epub 2005/11/30. doi: 23/34/8812 [pii] 10.1200/JCO.2005.03.1484 . 16314641

[78] De Preter K, Vandesompele J, Hoebeeck J, Vandenbroecke C, Smet J, Nuyts A, et al No evidence for involvement of SDHD in neuroblastoma pathogenesis. BMC cancer. 2004;4:55 . 15331017 10.1186/1471-2407-4-55 PMC517501

[79] Astuti D, Morris M, Krona C, Abel F, Gentle D, Martinsson T, et al Investigation of the role of SDHB inactivation in sporadic phaeochromocytoma and neuroblastoma. British journal of cancer. 2004;91(10):1835–41. . 15505628 10.1038/sj.bjc.6602202 PMC2410049

[80] Dickinson A, Yeung KY, Donoghue J, Baker MJ, Kelly RD, McKenzie M, et al The regulation of mitochondrial DNA copy number in glioblastoma cells. Cell Death Differ. 2013;20(12):1644–53. Epub 2013/09/03. doi: cdd2013115 [pii] 10.1038/cdd.2013.115 23995230 PMC3824586

[81] Facucho-Oliveira JM, John JC St. The relationship between pluripotency and mitochondrial DNA proliferation during early embryo development and embryonic stem cell differentiation. Stem Cell Rev. 2009;5(2):140–58. Epub 2009/06/13. 10.1007/s12015-009-9058-0 . 19521804

[82] Igney FH, Krammer PH. Death and anti-death: tumour resistance to apoptosis. Nat Rev Cancer. 2002;2(4):277–88. Epub 2002/05/11. 10.1038/nrc776 . 12001989

[83] Dey R, Moraes CT. Lack of oxidative phosphorylation and low mitochondrial membrane potential decrease susceptibility to apoptosis and do not modulate the protective effect of Bcl-x(L) in osteosarcoma cells. J Biol Chem. 2000;275(10):7087–94. Epub 2000/03/04. . 10702275 10.1074/jbc.275.10.7087

[84] Shidara Y, Yamagata K, Kanamori T, Nakano K, Kwong JQ, Manfredi G, et al Positive contribution of pathogenic mutations in the mitochondrial genome to the promotion of cancer by prevention from apoptosis. Cancer research. 2005;65(5):1655–63. Epub 2005/03/09. 10.1158/0008-5472.can-04-2012 . 15753359

[85] Kwong JQ, Henning MS, Starkov AA, Manfredi G. The mitochondrial respiratory chain is a modulator of apoptosis. The Journal of cell biology. 2007;179(6):1163–77. Epub 2007/12/19. 10.1083/jcb.200704059 18086914 PMC2140029

[86] Kim JY, Kim YH, Chang I, Kim S, Pak YK, Oh BH, et al Resistance of mitochondrial DNA-deficient cells to TRAIL: role of Bax in TRAIL-induced apoptosis. Oncogene. 2002;21(20):3139–48. Epub 2002/06/26. 10.1038/sj.onc.1205406 . 12082629

[87] King A, Selak MA, Gottlieb E. Succinate dehydrogenase and fumarate hydratase: linking mitochondrial dysfunction and cancer. Oncogene. 2006;25(34):4675–82. Epub 2006/08/08. doi: 1209594 [pii] 10.1038/sj.onc.1209594 . 16892081

[88] Hughes SD, Kanabus M, Anderson G, Hargreaves IP, Rutherford T, Donnell MO, et al The ketogenic diet component decanoic acid increases mitochondrial citrate synthase and complex I activity in neuronal cells. Journal of neurochemistry. 2014 Epub 2014/01/05. 10.1111/jnc.12646 . 24383952

