# Supplementary figures and images for: Inhibition of Neuroblastoma Tumor Growth by Ketogenic Diet and/or Calorie Restriction in a CD1-Nu Mouse Model
Source: PLoS One. 2015 Jun 8;10(6):e0129802. doi: 10.1371/journal.pone.0129802 (PMC4459995; doi:10.1371/journal.pone.0129802)

**S1 Figure** Activities of mitochondrial OXPHOS complexes I-V of SH-SY5Y xenograft tumors.

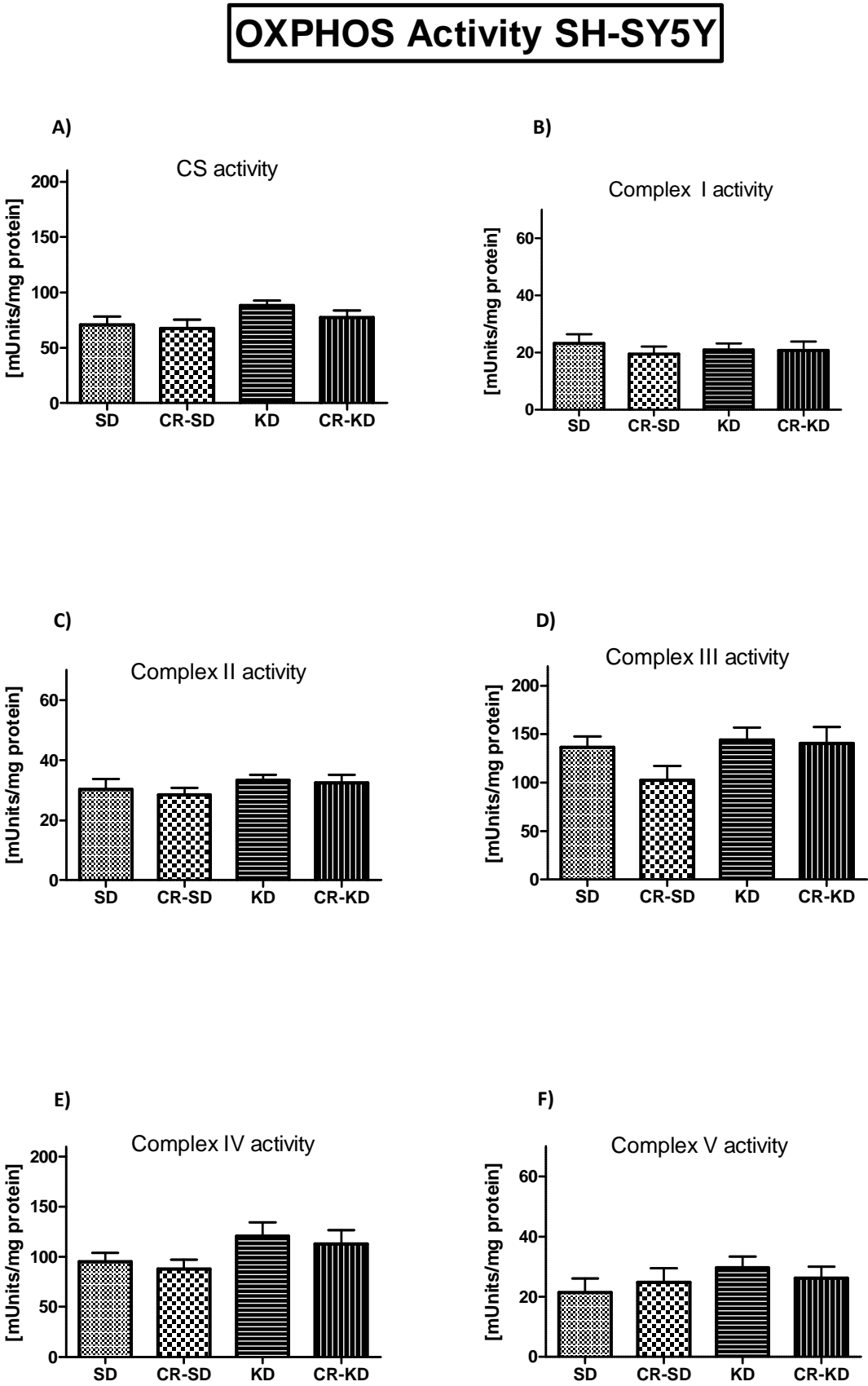

Supplement: S1 Fig — (PDF) [file pone.0129802.s001.pdf]

**S2 Figure** Activities of mitochondrial OXPHOS complexes I-V of SK-N-BE(2) xenograft tumors.

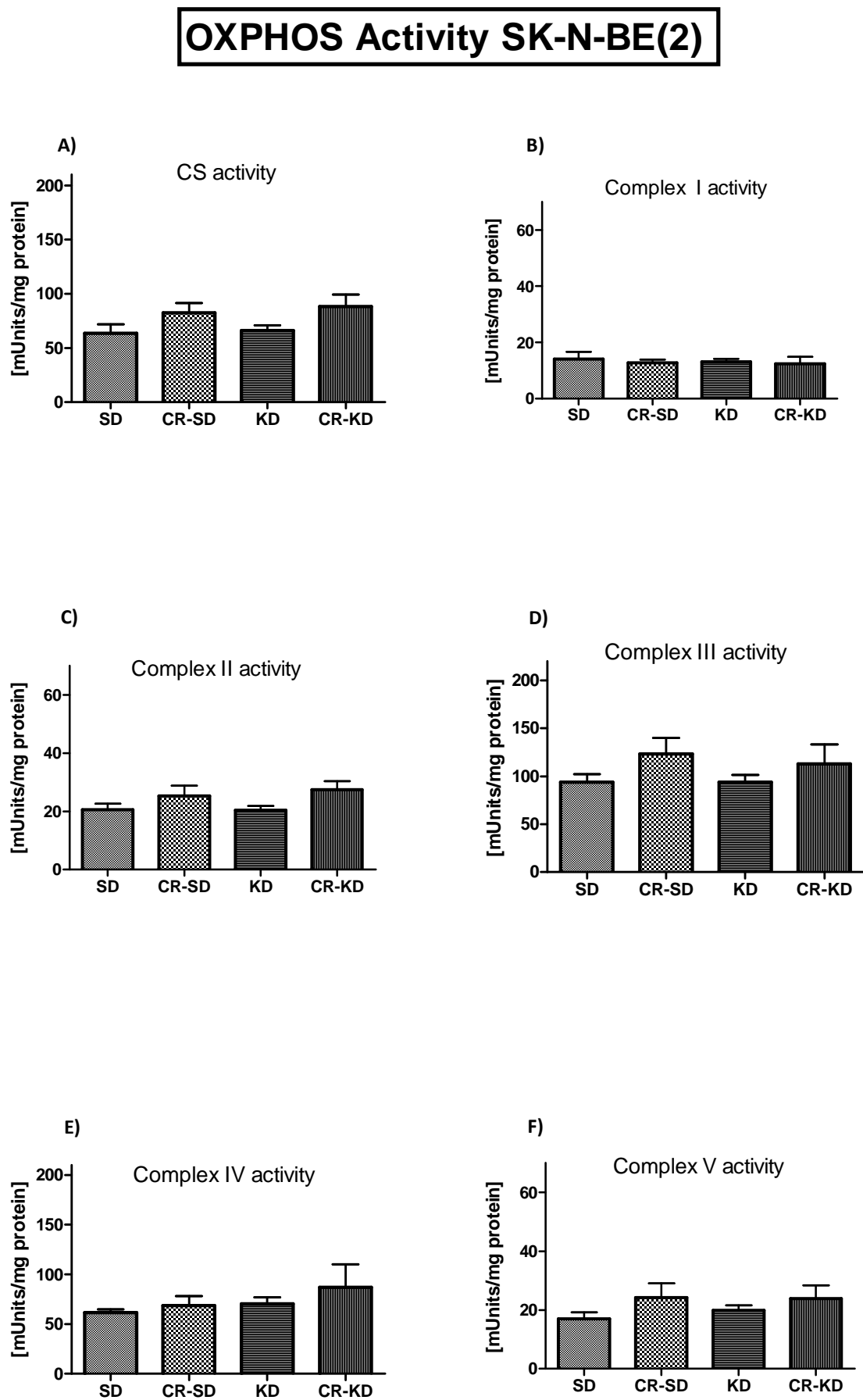

Supplement: S2 Fig — (PDF) [file pone.0129802.s002.pdf]

**S3 Figure** Westernblot analysis of SCOT expression in NB xenografts.

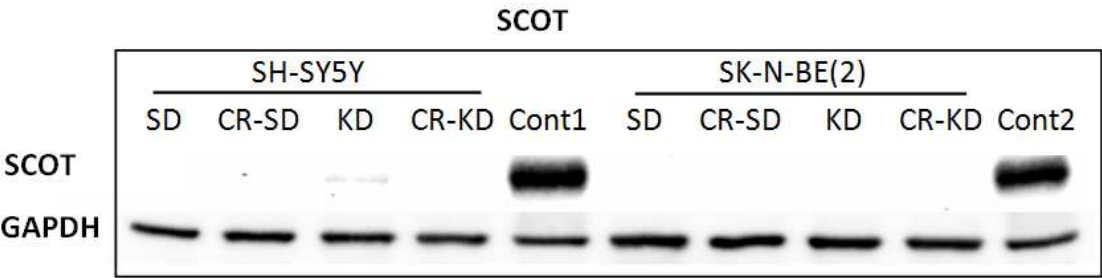

Supplement: S3 Fig — (PDF) [file pone.0129802.s003.pdf]

**S4 Figure** Westernblot analysis of SCOT expression in NB xenografts.

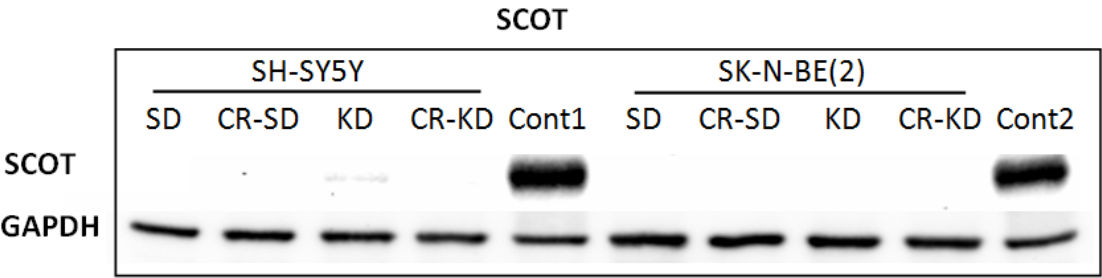

Supplement: S4 Fig — (PDF) [file pone.0129802.s004.pdf]
