# Supplementary material for: Inhibition of Neuroblastoma Tumor Growth by Ketogenic Diet and/or Calorie Restriction in a CD1-Nu Mouse Model
Source: PLoS One. 2015 Jun 8;10(6):e0129802. doi: 10.1371/journal.pone.0129802 (PMC4459995; doi:10.1371/journal.pone.0129802)
Supplement: S1 Table — (PDF) [file pone.0129802.s005.pdf]

**S1 Table** Ingredient list of the two diets.

| | Standard Diet | Ketogenic Diet |
|---|---|---|
| Crude Nutrients | % | % |
| Dry matter | 87.8 | 98.0 |
| Crude protein | 19.0 | 15.9 |
| Crude Fat | 3.3 | 57.0 |
| Crude fibre | 4.9 | 4.6 |
| Crude ash | 6.4 | 5.2 |
| N free extracts | 54.2 | 15.5 |
| Carbohydrates | 41.3 | 11.1 |
| Starch | 36.6 | 0.1 |
| Saccharose | 4.7 | 11.0 |
| | | |
| Fatty Acids | % | % |
| short chain | - | 0.19 |
| medium chain | - | 0.69 |
| long chain | 3.33 | 53.69 |
| | | |
| Minerals | % | % |
| Calcium | 1.00 | 0.92 |
| Phosphorus | 0.70 | 0.61 |
| Sodium | 0.24 | 0.19 |
| Magnesium | 0.23 | 0.21 |
| Potassium | 0.92 | 0.95 |
| | | |
| Amino Acids | % | % |
| Lysine | 1.10 | 1.32 |
| Methionine | 0.35 | 0.59 |
| Met+Cys | 0.70 | 0.86 |
| Threonine | 0.68 | 0.70 |
| Tryptophan | 0.25 | 0.21 |
| Arginine | 1.16 | 0.62 |
| Histidine | 0.45 | 0.48 |
| Valine | 0.90 | 1.11 |
| Isoleucine | 0.78 | 0.90 |
| Leucine | 1.33 | 1.58 |
| Phenylalanine | 0.87 | 0.82 |
| Phe+Tyr | 1.47 | 1.66 |
| Glycine | 0.82 | 0.34 |
| Glutamic acid | 3.97 | 3.58 |
| Aspartic acid | 1.66 | 1.18 |
| Proline | 1.28 | 1.82 |
| Alanine | 0.81 | 0.53 |
| Serine | 0.92 | 0.95 |
| | | |
