# Supplementary material for: Inhibition of Neuroblastoma Tumor Growth by Ketogenic Diet and/or Calorie Restriction in a CD1-Nu Mouse Model
Source: PLoS One. 2015 Jun 8;10(6):e0129802. doi: 10.1371/journal.pone.0129802 (PMC4459995; doi:10.1371/journal.pone.0129802)
Supplement: S2 Table — (PDF) [file pone.0129802.s006.pdf]

S2 Table MtDNA copy number primer list.

	Gene (s)	Forward primer	Reverse primer
mitochondrial fragments	MT-CO1	CGG AGG AGG AGA CCC CAT TC	TGG TAG CGG AGG TGA AAT ATG C
	MT-CO3, MT-TG, MT-ND3	AGC CGC CGC CTG ATA CTG	GGG GAT ATA GGG TGG AAG CCG
nuclear fragments	POLG	TCC TGT GGT CAT TTA TGG CA	TAG ATC CTG CCC ACC CAA G
	RRM2B	GCG ATA ATG CTG ATG TCC AG	CAT AAC CAA GCC GTA AGC AA
